# Recent Advances in Functional Nanomaterials for Enhancing Biopolymer-Based Active Food Packaging: A Review

**DOI:** 10.3390/gels11110905

**Published:** 2025-11-11

**Authors:** Rui Zhang, Chuanhuan Liu, Congyu Lin, Hong Zhang, Longwei Jiang, Yingzhu Liu

**Affiliations:** 1 School of Forestry and Landscape Architecture, Anhui Agricultural University, Hefei 230036, China; 2 School of Food Science and Technology, Jiangnan University, Wuxi 214122, China; 3 College of Mechanical Electrification Engineering, Tarim University, Alar 843300, China; 4 Key Laboratory of Jianghuai Agricultural Product Fine Processing and Resource Utilization of Ministry of Agriculture and Rural Affairs, Anhui Engineering Research Center for High Value Utilization of Characteristic Agricultural Products, College of Food and Nutrition, Anhui Agricultural University, Hefei 230036, China

**Keywords:** biopolymers, nanomaterials, food packaging, applied research, chemical classification

## Abstract

Food packaging serves a pivotal role in daily life, facilitating the efficient transportation of food and extending its shelf life. Petroleum-derived plastic packaging is extensively employed; however, its non-biodegradable nature poses significant environmental pollution and ecological degradation. Natural polymers (e.g., proteins such as gelatin and corn gluten protein; polysaccharides including pectin, chitosan, starch, cellulose, and alginate) and synthetic polymers (e.g., polyvinyl alcohol, polylactic acid, and polyhydroxyalkanoates) can be utilized to fabricate food packaging films, thereby achieving green and eco-friendly objectives. Nevertheless, the inferior mechanical strength and inadequate antibacterial activity of biopolymer-based packaging have restricted their practical applications. In recent years, nanomaterials (e.g., nanoparticles, nanotubes, nanofibers, and nanosheets) have been employed to enhance the performance of food packaging, emerging as a research hotspot. Notably, nanoparticles possess unique properties, including a high specific surface area, excellent dispersibility, and multifunctionality, which enables them to be easily incorporated into film matrices. Owing to their unique chemical structures, nanoparticles form strong interactions with film matrices, leading to a denser spatial structure. This not only markedly enhances the mechanical strength of the films, but also simultaneously improves their antibacterial and antioxidant capabilities. This review classifies and summarizes common nanomaterials based on their chemical compositions, providing a theoretical foundation and technical reference for the future development and application of nanomaterials in the field of bio-based active food packaging.

## 1. Introduction

Food storage has long been a persistent challenge in the food industry. Over prolonged storage periods, oxidative deterioration and microbial proliferation in food products not only compromise nutritional quality but also pose significant risks to human health, while simultaneously leading to substantial resource wastage. Food packaging serves as a critical barrier against contamination, adverse environmental factors, and physical damage, thereby safeguarding food quality and safety and playing an indispensable role in meeting consumer demands [1]. However, the majority of packaging materials currently available on the market are petroleum-derived polymers, such as polyethylene (PE) and polyvinyl chloride (PVC). Although the low cost and favorable processability of these plastics cater to economic considerations, their inherent poor biodegradability results in their accumulation in soil and marine ecosystems, causing long-term environmental pollution. Concurrently, microplastics generated from the degradation of such packaging materials permeate the human body via the food chain and drinking water sources, posing latent threats to human health. Furthermore, the production of petroleum-based packaging relies heavily on fossil fuel consumption and emits substantial amounts of greenhouse gases (e.g., carbon dioxide), which contribute to global warming and exacerbate climate change [2].

To address these challenges, research attention has shifted from non-degradable petroleum-based packaging materials toward biodegradable biopolymer-based packaging. As the name implies, biopolymers refer to natural or synthetic polymers derived from sustainable, renewable, and biodegradable feedstocks, typically sourced from plant- and animal-based resources [3]. Common biopolymers include natural biomacromolecules such as proteins (e.g., gelatin, zein), polysaccharides (e.g., pectin, chitosan, cellulose, starch, and alginates), as well as synthetic biopolymers (e.g., polyvinyl alcohol, PVA. polylactic acid, PLA. polyhydroxyalkanoates, PHA) [4]. Biopolymers possess excellent film-forming ability, versatile functional properties, and inherent biodegradability, thereby minimizing environmental footprint [5]. Furthermore, certain biopolymer molecules naturally exhibit antibacterial and antioxidant activities, which can enhance the preservative efficacy of food packaging materials [6].

**Figure 1:**
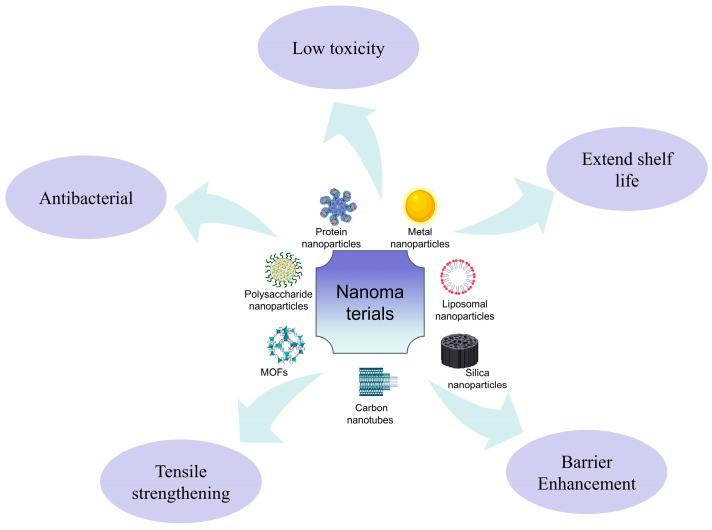
Functional properties of nanomaterials.

Compared to commercially available packaging materials currently employed, the application of biopolymers in food packaging still confronts several inherent limitations, such as inadequate antimicrobial activity, inferior mechanical properties, and high gas/water vapor permeability [6]. The incorporation of nanomaterials (e.g., nanoparticles, nanotubes, and nanofibers) into biopolymer matrices can modulate their intrinsic properties [7], while simultaneously regulating gas transport behavior within the film. During food storage, the accumulation of undesirable metabolic gases compromises the shelf life of food products [8]. Under these circumstances, nanomaterials exert a pivotal role in alleviating these adverse impacts. As illustrated in Figure 1, the integration of biopolymer-based technology and nanomaterials has paved a novel pathway for the advancement of active food packaging systems.

**Figure 2:**
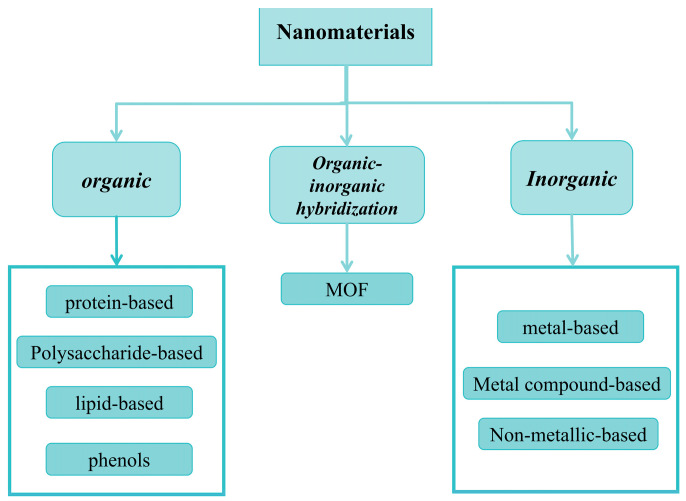
Classification of nanomaterials.

Research on the application of nanomaterials in food packaging has been extensively conducted. However, most of the studies are focused on a specific type of nanomaterial [9,10,11] and on a particular film substrate, exploring the use of different nanomaterials [12]. A few studies, on the other hand, classify and describe nanomaterials based on their different functionalities [13,14]. This review differs from other studies in that it classifies nanomaterials from a new perspective based on their chemical composition and structural characteristics. As show in Figure 2, this classification method is more detailed and precise, and can be tailored for specific uses in different application scenarios, which is beneficial for the future development and utilization of nanomaterials in the field of active food packaging.

## 2. Organic Nanomaterials

Organic nanomaterials feature a carbon-based core framework and are predominantly derived from natural polymers or biodegradable synthetic materials. Their chemical structure exhibits high similarity to that of bio-based substrates, which facilitates the formation of a homogeneous blended system with the substrate—free from the risk of interfacial delamination. Furthermore, these nanomaterials are fully biodegradable, and their degradation products such as glucose and amino acids are non-toxic, aligning with the “edible-grade” safety standards for food packaging. These attributes collectively render organic nanomaterials a prominent research focus in the field of active packaging.

### 2.1. Protein-Based

Protein-based nanomaterials exhibit distinct advantages, including excellent mechanical extensibility and facile conjugation with active substances (e.g., antimicrobial agents and antioxidants). Common proteins employed for fabricating these nanomaterials include zein and sericin.

Gelatin, a water-soluble macromolecular protein, exhibits thickening, gelling, and stabilizing capabilities. Owing to its superior film-forming capacity, it serves as one of the primary materials for biopolymer-based packaging. However, its inferior mechanical strength, inadequate barrier properties, and high susceptibility to microbial contamination restrict its practical applications in food packaging [10]. Incorporation of functional nanoparticles can mitigate these limitations. Zein, a self-assembling and biocompatible protein, is extensively utilized for constructing food-grade nanoparticles, thereby further enhancing the physical stability of the system [15]. Bacterial cellulose is a biopolymer that can be synthesized and has excellent film-forming ability, but it lacks antibacterial and antioxidant properties. Adding silymarin with excellent antibacterial and antioxidant activities to bacterial cellulose nanofiber films is a challenge due to their poor water solubility. However, this problem has been solved by loading silymarin into Zein to prepare nanoparticles. The preparation of composite nanoparticles has enhanced the solubility of silymarin, and the film has shown better preservation ability, thereby extending the shelf life of salmon [16]. Multilayer nanoparticles composed of curcumin–resveratrol–chondroitin sulfate combined with corn alcohol-soluble protein were modified using atmospheric cold plasma (ACP) and incorporated into gelatin films [17]. Enhanced hydrogen bonding and electrostatic interactions between nanoparticles and the gelatin matrix significantly improved the film’s mechanical strength. Oxygen permeability (OP) decreased from 3.61 ± 0.05 g/m·d to 1.82 ± 0.03 g/m·d, while water vapor permeability (WVP) dropped from (7.45 ± 0.76) × 10^−7^ g/m·d·Pa to (2.63 ± 0.86) × 10^−7^ g/m·d·Pa. The barrier properties of the gelatin film improved, with DPPH and ABTS scavenging rates increasing from 29.38 ± 1.59% and 45.33 ± 2.34% to 47.62 ± 2.09% and 61.88 ± 1.13%, respectively. For refrigerated preservation of fish fillets, unpackaged samples showed spoilage on day 4, while those wrapped in polyethylene (PE) film or pure gelatin film delayed spoilage until day 6. Notably, the incorporation of nanoparticles further prolonged the spoilage onset to day 8. Additionally, resveratrol-loaded zein/pectin nanoparticles were incorporated into loquat seed starch-based films. The presence of these nanoparticles facilitated robust hydrogen bonding between resveratrol and the film matrix, remarkably enhancing the antioxidant activity, barrier performance, and tensile strength, while reducing the light transmittance and moisture content of the films. This effectively inhibited the oxidation of soybean oil during storage, thereby providing superior protective effects for fatty food packaging [18].

Silk fibroin (SF) also serves as a primary raw material for protein-based nanomaterials. Using SF to encapsulate eugenol (EO) through self-assembly yields nanoparticles that effectively mask EO’s pungent odor while enhancing stability. Incorporating these nanoparticles into goji berry polysaccharide nanofibers via electrospinning yields more stable fiber films with finer, more uniform diameters. Compared to fiber films without nanoparticles, the inhibition zones against *Escherichia coli* and *Staphylococcus aureus* increased from 10 mm to 19 mm. The preservation effect on pork was also significantly better than the control group, and the addition of nanoparticles did not negatively affect the color or texture of the pork [19].

### 2.2. Polysaccharide-Based

The core advantages of polysaccharide-based materials lie in their inherent antibacterial activity, excellent barrier properties (water and oxygen resistance), and renewability. Among polysaccharide-based nanomaterials used in food packaging, chitosan and cellulose are the most prevalent.

Chitosan (CS) is a naturally occurring positively charged compound with biocompatibility, degradability, and non-toxicity [20]. It has been demonstrated that bulk chitosan can be fabricated into nanoparticles—specifically chitosan nanoparticles (CNPs) with sizes ranging from 1 to 100 nm [21]. CS-derived nanoparticles show significant potential in nanomaterial applications, including as antibacterial agents, drug delivery systems, and bioimaging probes [22]. Four primary methods for preparing chitosan nanoparticles are ionogelation, microemulsion, emulsified solvent diffusion, and polyelectrolyte complexation [23]. Among these, ionogelation with negatively charged anionic crosslinkers is the simplest and most widely used synthesis method [24]. Common crosslinking agents include phytic acid, glutaraldehyde, and tripolyphosphate [25]. Notably, although CNPs exhibit antibacterial activity, they only act on bacteria in direct contact with the particles. This limitation restricts their application in biopolymer-based active food packaging [26]. Consequently, researchers have incorporated volatile active agents to achieve synergistic antibacterial effects. Specifically, CS (cationic) and rhamnolipid (anionic) were combined to prepare nanoparticles loaded with chlorogenic acid, which were then incorporated into branched starch–gelatin composite films. These composite films exhibited excellent antibacterial activity and outstanding free radical scavenging capacity. Toxicity tests confirmed the good biocompatibility of the nanoparticle-incorporated composite films, which also showed superior preservation effects for bananas and chicken compared to PE films and starch–gelatin composite films without nanoparticles [27]. Additionally, research has utilized ionogelation to synthesize CNPs from chitosan and sodium tripolyphosphate (TPP). A bionanocomposite film was prepared by blending thymol (Thy), CNPs, and corn starch. Due to crosslinking between chitosan and TPP, the CNPs exhibited small sizes and regular morphologies, which effectively filled voids in the starch matrix. This resulted in a denser film structure with significantly enhanced mechanical properties: tensile strength increased from 7.9 MPa to 13.7 MPa, while water vapor permeability decreased. Owing to the synergistic antibacterial effects of CNPs and Thy, the shelf life of cherry tomatoes was extended from 2 days to 6 days [26].

Cellulose is a common polysaccharide-based natural polymer characterized by low density, strong mechanical properties, and large surface area. Possessing potential for modification, non-toxicity, and biodegradability, it is frequently used as a raw material for polysaccharide-based nanomaterials. Cellulose nanofibers (CNFs) are obtained through physical, chemical, or biological methods such as high-pressure homogenization, carboxymethylation, and acid hydrolysis [28], and cellulose nanofibers (CNFs) incorporated into active packaging can enhance physical, mechanical, and barrier properties [29]. Research has utilized ionic liquids as solvents to prepare mechanically strengthened composite ionic gels by combining carbon nanotubes (CNTs) and polyvinyl alcohol (PVA). These gels resist fracture under distortion, with a 1 mm thick gel capable of supporting a 1.5 kg load [30].

### 2.3. Lipid-Based

Lipid-based nanomaterials, unlike protein- or polysaccharide-based nanomaterials which are constrained by thermal processing conditions, possess higher potential for industrial-scale production and can protect active substances against environmental factors (e.g., pH, enzymes, and oxygen) [31]. These include nanoliposomes (NLs) and nanostructured lipid carriers (NLCs). Representative types include nanoliposomes (NLs) and nanostructured lipid carriers (NLCs) [32]. NLs are spherical lipid vesicles composed of two or more bilayers [31], with phospholipids as the core lipids. They exhibit high biocompatibility, non-immunogenicity, and non-toxicity [33]. Encapsulation of garlic essential oil via NLs, followed by incorporation into chitosan films, increased the antioxidant activity from 33.5% to 50.93% [34]. When applied to chicken strip packaging, this composite film effectively inhibited the formation and degradation of amines and nitrogen-containing compounds, thereby extending the shelf life by 2–3-fold. Common methods for NL preparation include mechanical stirring, ultrasonication, extrusion, and microfluidization. However, limitations such as high production costs, poor physical stability, and low active substance loading capacity restrict their practical applications [35].

NLCs are composed of solid lipids supplemented with a small amount of liquid lipids, and contain almost no phospholipids [36]. Solid lipids offer higher density and superior stability; since active substances are encapsulated within the solid matrix, their release is more gradual and controllable. Furthermore, the wide availability of solid lipid sources contributes to the lower production cost of NLCs. Common preparation methods include ultrasonication, solvent diffusion, and homogenization [37].

### 2.4. Phenols

Phenolic nanomaterials are a class of nanomaterials composed of aromatic polymers abundant in phenolic hydroxyl groups, exhibiting outstanding antioxidant activity and UV-shielding properties. Primary preparation methods include molecular self-assembly, nanoprecipitation, and polymerization. Their core structure consists of a phenolic ring with attached phenolic hydroxyl groups, endowing them with unique π-π stacking and metal coordination capabilities that distinguish them from the other three categories of organic nanomaterials [38].

Lignin contains functional groups such as aromatic, phenolic, methoxy, and carboxylic acid groups and exhibits antibacterial, antioxidant, and UV-blocking properties [39]. Its highly variable chemical structure endows it with enhanced reactivity when fabricated into nanoparticles [40]. Lignin nanoparticles (LNPs) extracted from sugarcane bagasse were incorporated into cellulose nanofibril (CNF) films [41]. Although the mechanical properties and water vapor barrier performance of the films exhibited no significant improvements, their UV-shielding capability was markedly enhanced—an effect attributed to the non-conjugated phenolic groups in lignin [42]. Simultaneously, the controlled release of lignin from the films acted as the primary radical-scavenging mechanism. The incorporation of LNPs also inhibited microbial growth, extending the shelf life of raspberries packaged with CNF/LNP films from 2 days to 10 days relative to commercial plastic packaging. Additionally, LNPs synthesized using bamboo powder were incorporated into casein films, which significantly enhanced the films’ mechanical properties [43]. Compared to pure casein films, the tensile strength increased by 219.7%, water solubility decreased from 31.65% to 24.81%, and both antibacterial and antioxidant properties were remarkably improved. Consequently, the shelf life of strawberries was extended from 6 days (with pure casein films) to 9 days. The composite films underwent complete degradation within 45 days, and the incorporation of LNPs did not exert a negative impact on film degradability.

Tannic acid (TA) is a natural polyphenolic molecule that can be extracted from various plants such as green tea, grapes, and oak trees [44]. Due to its strong adhesive properties, it frequently interacts with other polymeric materials [4]. Nanoparticles prepared by depositing TA onto lignin surfaces and incorporated into polyvinyl alcohol films achieved 99.4% UV shielding capability. Tensile strength increased from 31.42 to 37.38 MPa, while banana shelf life extended from 3 to 6 days before skin browning occurred [45]. Due to the presence of phenolic hydroxyl groups, stable metal ion-crosslinked tannic acid (MITA) nanostructures can also form through coordination bonds with metal ions. By self-assembling Cu^2+^ and TA, Cu@TA NPs were synthesized that can be firmly anchored to bacterial films. Through the release of Cu^2+^ and TA, these particles completely inhibit the growth of both Gram-positive and Gram-negative bacteria, exhibiting synergistic antibacterial effects [46].

To conclude, this organic nanomaterial, owing to its green and non-toxic nature, exhibits excellent biocompatibility. It can form intimate interactions with food packaging matrices, achieving uniform dispersion via chemical bonding and physical interactions. Ultimately, it undergoes complete biodegradation, which is consistent with the prevailing focus on green and environmental sustainability. Meanwhile, the advancement and maturity of processing technologies have addressed the existing bottleneck in large-scale production. The organic nanomaterial itself possesses inherent antibacterial and antioxidant properties and can be integrated with other active agents to synergistically improve food preservation efficacy. Its application scope is extensive, encompassing fruits, vegetables, and meat products, as summarized in Table 1. However, owing to the presence of abundant hydrophilic groups in their molecular structure, organic nanomaterials exhibit poor resistance to moisture—rendering them unsuitable for packaging high-humidity-sensitive food products. Additionally, they are highly susceptible to environmental factors such as temperature, light, and humidity and prone to degradation or performance deterioration during storage or application, whereas most organic nanomaterials possess limited intrinsic antibacterial and antioxidant activities, relying on the loading of external active substances to achieve desired functional effects. Notably, their relatively high production costs, driven by complex preparation and purification procedures, continue to constrain large-scale application in food-packaging systems. Although the raw materials themselves are non-toxic, concerns persist regarding their long-term safety, such as potential intestinal accumulation. Currently, relevant data remain insufficient, necessitating further in-depth investigations.

## 3. Inorganic Nanomaterials

Inorganic nanomaterials have found extensive application in active food packaging due to their physical, chemical, antimicrobial, optical, and mechanical properties, thermal stability, low-cost production, safe usage, and compatibility with organic compounds [47]. They are commonly incorporated into bio-based films to enhance mechanical properties and antimicrobial performance. Due to differences in application scenarios, it is of great significance to select appropriate inorganic nanomaterials. The comparison among them is shown in Table 2.

### 3.1. Metal-Based

Metal-based nanomaterials primarily consist of metallic elements (such as gold, silver, copper, zinc) or alloys, exhibiting outstanding antibacterial properties. Their antibacterial mechanism involves releasing positively charged ions that act as antimicrobial agents—these ions interact with bacterial cell films, causing film rupture. This leads to the release of intracellular substances, ultimately rendering bacterial cells inactive and resulting in their death [56]. In addition to their excellent antibacterial properties, the presence of metal ions also enables the adsorption and decomposition of harmful components in food packaging, such as oxygen, moisture, and odors, effectively extending the shelf life of food products [57]. Current research on metal-based nanomaterials for active food packaging primarily focuses on silver, with relatively limited exploration of other metals such as gold and copper, as shown in Table 3.

Silver nanoparticles (AgNPs) not only enhance the mechanical properties and bioactivity of biopolymer packaging films but also impart excellent UV-barrier and photothermal properties to the films, attributed to their unique surface plasmon resonance (SPR) effect [66]. Additionally, AgNPs exhibit a strong affinity for sulfur- and nitrogen-containing biomolecules, forming various ligand bonds (e.g., Ag-N and Ag-S). These bonds interact with bacterial biomolecules, thereby inhibiting bacterial growth and proliferation and conferring outstanding antibacterial activity [67]. Lichen polysaccharide–silver nanoparticles (L-AgNPs), green-synthesized using lichen polysaccharide, were incorporated into chitosan-based films, resulting in a 138.8% increase in tensile strength. For the preservation of fresh-cut apple slices, the onset of browning was delayed relative to PE films under identical storage conditions. Notably, the incorporation of AgNPs endowed the films with exceptional photothermal conversion properties: when supplemented with near-infrared irradiation, the films achieved sterilization rates exceeding 95% against both *Escherichia coli* and *Staphylococcus aureus*, demonstrating a superior antimicrobial performance [68]. The incorporation of AgNPs into nanofiber (NF)–carrageenan films reduced the transparency of the composite films due to the presence of metal ions [69]. However, the tensile strength increased from 3.18 ± 0.63 MPa to 6.81 ± 0.46 MPa, while the elongation at break increased from 9.23 ± 1.98 mm to 18.73 ± 1.9 mm. Storage tests on bread showed that samples packaged in plastic developed mold after 7 days, whereas bread packaged in the composite carrageenan films remained in good condition after 28 days. Silver migration assays on the composite films revealed a migration concentration of 0.013 μg/g, significantly below the maximum permissible limit of 0.05 μg/g stipulated by EU Regulation No. 10/2011 [70], confirming their safety.

Despite the immense potential of metal-based nanomaterials in active food packaging, most countries have yet to establish clear migration limits and testing standards for metallic nanomaterials in food packaging. It is believed that with the continuous advancement of nanotechnology and the ongoing refinement of relevant regulatory frameworks, metal-based nanomaterials will enjoy broader development prospects [9].

### 3.2. Metal-Compound-Based

Metal-compound-based nanomaterials are a class of inorganic materials formed at the nanoscale by combining metal elements with non-metal elements (primarily oxygen) through chemical bonds (ionic, covalent, or hybrid bonds). Compared to metal-based nanomaterials, the incorporation of non-metal compounds reduces the leaching rate of metal ions, resulting in lower toxicity. This makes them more suitable for packaging applications involving direct food contact. Metal oxides possess antibacterial properties, scavenge oxygen and ethylene, and exhibit excellent thermal stability, making them increasingly applicable in active food packaging [71]. In recent years, researchers have shown significant interest in metal oxide nanoparticles (MOMPs), such as TiO_2_, ZnO, and NiO [72].

Nickel oxide (NiO) is renowned for its antibacterial properties and biocompatibility. Incorporating it into nanofibers enhances their performance [65]. Researchers employed plant leaf extracts to green-synthesize nickel oxide nanoparticles (NiONPs). Incorporating these into cellulose acetate nanofibers via electrospinning increased tensile strength from 2.20 ± 0.42 MPa to 21.76 ± 1.15 MPa. Preservation experiments on lemons and tomatoes confirmed complete freshness retention for 10 days, whereas the control group exhibited spoilage by the third day [73].

Zinc oxide nanoparticles (ZnONPs) have emerged as prominent metal oxide nanoparticles, characterized by excellent chemical stability, UV-blocking capabilities, and antibacterial/antiviral activity [74]. As an essential trace element for the human body, zinc has been designated as “Generally Recognized as Safe” (GRAS) by the U.S. Food and Drug Administration (FDA) [75]. An antibacterial fiber film based on polylactic acid (PLA) and ZnONPs was fabricated via electrospinning technology [76], which enhanced the film’s thermal stability and increased its thermal degradation temperature from 175.17 °C to 258.67 °C. In contrast to pure PLA films without inherent antimicrobial activity, the ZnONP-incorporated composite film extended the refrigerated shelf life of chicken meat by 3 days. This effect was attributed to oxidative damage to bacteria induced by Zn^2+^ release and reactive oxygen species (ROS) [77].

TiO_2_ nanoparticles (TiO_2_NPs), featuring excellent antibacterial activity, UV-blocking properties, and photocatalytic performance [78], have been restricted in food packaging applications owing to the employment of chemical reagents in their conventional synthesis routes. Currently, researchers are focusing on developing non-toxic, eco-friendly synthesis methods to mitigate this limitation. Specifically, TiO_2_NPs synthesized using tea leaf extracts were incorporated into polyvinyl alcohol/chitosan/branched starch composite films, resulting in a denser internal network structure and consequently improved mechanical properties. Benefiting from the physical barrier effect of TiO_2_NPs, the oxygen transmission rate (OTR) decreased from 0.706 cc/(m^2^·day) to 0.307 cc/(m^2^·day). At a loading content of 1.5 wt%, the sterilization efficiency against *E. coli* attained 100%, thereby significantly enhancing the comprehensive performance of the composite films and expanding their application scope [79].

Magnesium oxide is a common metal oxide certified by the European Union as a food additive (E 530) due to its low toxicity. Its nanoparticles (MgONPs) exhibit outstanding antibacterial properties by releasing superoxide anions (O_2_^−^). When 1% MgONPs were incorporated into pectin films for cherry tomato preservation, the nanoparticle addition reduced the film’s permeability. This prevented carbon dioxide leakage from the packaging interior while blocking oxygen ingress from the external environment, thereby decreasing cherry tomato respiration and effectively extending shelf life [80].

### 3.3. Non-Metallic-Based

Non-metallic nanomaterials are inorganic nanomaterials that contain no metallic elements, with non-metallic components as the core, and leverage nanoscale effects to realize their functionalities. Compared with metal-based and metal-compound-based nanomaterials, they exhibit higher biocompatibility and eliminate the risk of metal ion release. Additionally, their reliance on non-metallic covalent bonds endows them with higher bond energy and enhanced chemical stability, rendering them less susceptible to extreme environmental conditions. Furthermore, due to their low cost, high availability, and eco-friendly characteristics, they are increasingly utilized in biopolymer-based active food packaging.

Among polysaccharide-based films, pectin is a primary material attributed to its high compatibility, commercial accessibility, and low cost. Pectin is predominantly composed of α-D-galacturonic acid, along with side chains of galactose, arabinose, and rhamnose [81]. Pure pectin films exhibit inadequate antibacterial activity; moreover, their weak mechanical strength and high hydrophilicity further restrict their practical applications in food packaging. Selenium nanoparticles (SeNPs) can inhibit the growth of foodborne pathogens while possessing UV-shielding properties [82]. Upon the incorporation of SeNPs into pectin films with Ca^2+^ as a crosslinking agent, the tensile strength of the pectin films increased from 9.11 MPa to 12.68 MPa, while the elongation at break (flexibility) rose from 10.58% to 25.18%. Consequently, the shelf life of fresh strawberries was extended from 3 days to 7 days [83].

Carbon quantum dots (CDs) have emerged as a promising material for active food packaging owing to their strong antioxidant and antibacterial properties, nanoscale size, and unique optical characteristics. Research indicates that incorporating CDs into pectin films enhances thermal stability. Due to the presence of CDs, composite films exhibited fluorescence under UV light, with tensile strength increasing from 3.62 ± 1.22 to 6.96 ± 1.45 MPa. The DPPH radical scavenging rate increased from 25% to 60%. Compared to the pure pectin film, which showed no antibacterial effect, the addition of CDs resulted in significant bacterial apoptosis, markedly enhancing the film’s antimicrobial properties. In strawberry preservation, unpackaged strawberry showed signs of rot by day 3, while those covered with pure pectin film began partial deterioration by day 5. In contrast, strawberries wrapped with the composite film retained their fresh color and showed no visible mold growth even after 5 days [84].

Silicon dioxide nanoparticles (SiNPs), as a porous nanomaterial, have attracted considerable attention owing to their large specific surface area, high pore volume, and excellent thermal stability [85]. Conventional synthesis approaches encompass thermal evaporation, chemical vapor deposition, biochemical synthesis, and hydrothermal synthesis [86]. To address the requirements of active food packaging, researchers have increasingly focused on more eco-friendly and cost-effective strategies. Specifically, SiNPs were prepared from rice bran via green synthesis, loaded with oak extract, and subsequently incorporated into a composite film based on papaya seed mucilage and alginate. This modification not only improved the film’s hydrophobicity and flexibility but also enhanced its antimicrobial activity, thereby extending the shelf life of pork by 20 days [87].

In summary, unlike organic nanomaterials, inorganic nanomaterials possess intrinsically high hardness and elastic modulus, enabling substantial reinforcement of bio-based films. Their dense structures effectively impede the permeation of gases and water vapor, making them particularly suitable for packaging foods that demand stringent barrier performance, such as fresh meat and ready-to-eat products. Inorganic nanomaterials also exhibit antibacterial and antioxidant activities through multiple mechanisms, including metal-ion release and reactive oxygen species (ROS) generation. Notably, they offer exceptional environmental stability, which supports processing under complex conditions (e.g., high-temperature extrusion) and ensures durability during long-term storage. In addition, more accessible raw materials and more mature manufacturing processes typically yield lower production costs, enhancing their compatibility with industrial-scale food-packaging applications.

Nonetheless, many inorganic nanomaterials exhibit limited biocompatibility. Their interactions with bio-based substrates are typically limited to physical adsorption, with little or no chemical bonding, which results in weak interfacial adhesion. During storage, migration of metal ions from such composite films into the food matrix may occur, posing potential safety risks; therefore, strict dosage control is necessary, and dedicated standards and regulations should guide their use. Furthermore, compared with organic nanomaterials, inorganic counterparts often provide relatively narrow functionality and cannot meet the “multifunctional synergies” required for bio-based active packaging without the addition of organic active components. In some cases, their activity also depends on external triggers—for example, TiO_2_ NPs require ultraviolet (UV) irradiation—so they cannot function autonomously in the absence of such stimuli.

## 4. Organic–Inorganic Hybrid Nanomaterials

Organic–inorganic hybrid nanomaterials are a class of nanoscale composites constructed via the integration of organic and inorganic components through chemical bonds or strong non-covalent interactions. In recent years, metal-organic frameworks (MOFs) have been increasingly utilized in active food packaging owing to their well-defined structures, exceptionally high porosity, large specific surface area, and superior stability [88]. MOFs are composed of metal ions (e.g., Co^2+^, Zr^4+^, Zn^2+^) linked by organic bridging ligands, featuring facile and eco-friendly synthesis routes [89]. Notably, it is crucial to differentiate MOFs from metal-based nanomaterials. Although MOFs contain metal ions, their functional mechanism involves the formation of hybrid frameworks via coordinate bonds. Their physicochemical properties are dominated by organic–inorganic synergistic effects, thus combining the functional advantages of organic materials with the structural stability of inorganic materials. This is fundamentally distinct from the metallic bonding-based systems in metal-based nanomaterials [90]. Via the incorporation of ZIF-67 nanoparticles into polylactic acid (PLA) fiber films using electrospinning technology, the tensile strength of a monolayer fiber film was tripled. Pure PLA films exhibited no inherent antimicrobial activity; however, upon ZIF-67 incorporation, Co^2+^ released from the nanoparticles interacted with bacteria, inducing bacterial death via a membrane-damaging mechanism. Inhibition zones with diameters of 4.4 ± 1.5 mm and 3.4 ± 1.2 mm were formed against *Escherichia coli* and *Staphylococcus aureus*, respectively. Compared to the pure PLA film, the ZIF-67-incorporated PLA film exhibited no rot or mold formation on citrus surfaces after 20 days of storage at 25 °C, demonstrating superior preservative properties [90].

Beyond their intrinsic antimicrobial activity, MOFs are also employed for the encapsulation and delivery of active substances owing to their well-defined internal pore structures, thereby synergistically augmenting antimicrobial efficacy. Among these, the zinc-based zeolitic imidazolate framework (ZIF-8) serves as a primary matrix for encapsulating trans-cinnamaldehyde (TC). When integrated into polyvinyl alcohol (PVA) films, the diameter of the inhibition zone against *Escherichia coli* increased from 0 to 18.33 mm. After wrapping spinach leaves with these composite films, enumeration of bacterial colonies on the leaves revealed that TC-loaded ZIF-8 nanoparticles significantly enhanced the films’ antimicrobial performance [91].

## 5. Material Application and Performance Evaluation

In the selection of nanomaterials, compatibility with the film polymer’s characteristics, core packaging requirements, and the suitability of the incorporation form must be prioritized. In scenarios where only mechanical performance needs enhancement or the nanomaterials themselves can fulfill packaging requirements—such as in polysaccharide-based packaging materials—natural nanomaterials or non-metallic-based nanomaterials homologous to the polymer matrix can be incorporated directly in a free form. This approach meets the packaging demands of short-term ready-to-eat foods while simplifying processing and reducing costs. When additional active ingredients are required to enhance the packaging’s preservative efficacy—for instance, in protein matrix packaging—encapsulation approaches are necessary. For example, in the context of long-term preservation of dairy products, lipid-based nanomaterials can be encapsulated within protein/polysaccharide carriers to satisfy the stringent safety requirements for food contact [92].

Furthermore, when nanomaterials themselves are sensitive to environmental factors or pose potential risks upon direct incorporation, carrier-mediated encapsulation is imperative. A case in point is photothermally sensitive phenolic nanomaterials, which, when encapsulated in chitosan carriers, can effectively preserve fruits [93]. For metal-based and metal-compound-based nanomaterials, encapsulation is recommended to mitigate the risk of metal ion migration, their incorporation into film matrices via encapsulated forms enables medium-to-long-term preservation of meat products. For synthetic polymer packaging characterized by high brittleness and poor barrier performance, incorporating organic nanomaterials in a free form is recommended to improve mechanical strength and enhance barrier properties, making it suitable for high-strength applications such as takeout containers. Furthermore, organic–inorganic hybrid nanomaterials can be integrated in an encapsulated form to impart antibacterial functionality, while preserving the inherent toughness of polylactic acid (PLA) [94], ensuring compatibility with polyhydroxyalkanoates (PHA) without compromising biodegradability [95]. This approach is appropriate for packaging pre-prepared food products

## 6. Industrialization and Challenges

**Figure 3:**
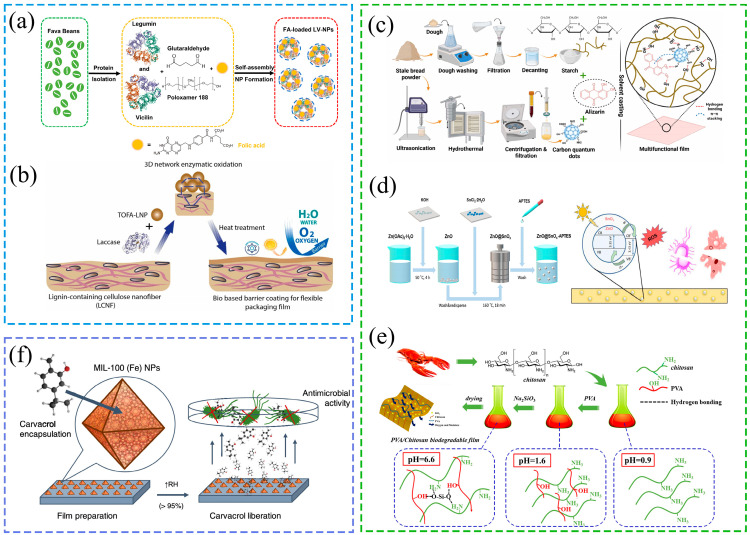
Mechanism of action regarding different nanomaterials. (**a**) Protein nanoparticles used for encapsulating folic acid [97]. (**b**) Lignin nanoparticles are utilized for barrier packaging coatings [98]. (**c**) Development of CDs for smart food packaging [99]. (**d**) Tin oxide-coated zinc oxide nanocomposites are utilized for antibacterial applications in food packaging [100]. (**e**) Silicon nanomaterials are utilized to enhance the performance of food packaging [101]. (**f**) MOFs are utilized to prolong the duration of antibacterial activity [102].

The integrated application of nanostructures in packaging has achieved multidimensional implementation through industrial production: smart packaging integrating temperature regulation and traceability has entered partial mass production, while nanoscale sensor detection modules are being adapted for large-scale scenarios like cold chains; Active packaging incorporating functional molecules like antimicrobial agents has entered batch production, with core processes compatible with existing production lines, widely used for preserving meat, fruits, and vegetables; industrial formulations for nanocomposite coatings and substrates have been optimized and implemented, enhancing material physicochemical properties through scaled-up addition of nanofillers while maintaining production efficiency comparable to traditional processes; nanostructure-based carrier technology for nutrient delivery has seen small-scale application in functional foods, supporting the large-scale development of nutritionally fortified products [96]. Currently, the development of various nanomaterials is garnering widespread attention in the community, as illustrated in Figure 3.

However, with the rapid advancement of nanomaterials in food packaging, it has become increasingly evident that unlike traditional materials, nanomaterials can not only migrate from polymer matrices but also adsorb onto food surfaces, form agglomerates, or undergo structural transformations during interactions with food [103]. Migration behavior is regulated by physicochemical factors, including food composition, contact time and temperature, contact area, nanomaterial size, food pH, storage conditions, and nanomaterial concentration [104]. Of particular concern is the safety of metallic nanomaterials, thus requiring strict regulation of metal ion migration. Excessive migration may induce cytotoxicity, genotoxicity, or bioaccumulation, as these ions can penetrate cells and interact with intracellular organelles. The diverse physicochemical properties of nanomaterials render safety assessments more complex. Regulatory authorities such as European Food Safety Authority (EFSA) and the U.S. Food and Drug Administration (FDA) acknowledge these risks but have not yet established universally accepted testing protocols [105].

## 7. Conclusions and Outlook

Currently, extensive research efforts are underway to develop diverse nanomaterials for application in various active food packaging systems, which represents the prevailing trend in the future development of food packaging. Notably, the application of nanomaterials is also constrained by the costs of different polymer packaging materials. High-value natural polymer packaging materials (e.g., whey protein films and chitosan films) exhibit considerable costs; however, superior packaging performance can be attained through the simple integration of low-cost nanomaterials. In contrast, low-cost materials such as starch-based films are limited by their inherent functional deficiencies and thus require the integration of high-value nanomaterial technologies to meet practical application requirements. By comparison, synthetic polymer packaging materials (PLA, PHA, PVA) typically exhibit relatively lower costs, and the application of nanomaterials—especially nanocellulose (CNF/CNC) derived from agricultural wastes (e.g., sweet potato residue and rice husk)—significantly improves film performance when combined with PLA [106], primarily via hydrogen bonding and the formation of a dense network. Meanwhile, the cost of this composite system has approached that of traditional petroleum-based plastics, endowing it with promising economic substitution potential. With the support of policies and the improvement of the circular economy system in the future, nanomaterials technology by recovery and development from agricultural waste combined with PLA/PHA matrices will occupy the main position in food packaging.

The synergistic integration of nanomaterials and biopolymers represents a new developmental stage in food packaging technology. By incorporating the unique advantages of nanoscale effects into macroscopic packaging design, this technological combination has successfully surpassed the performance limitations of traditional packaging. Nanomaterials not only act as “reinforcing agents” to significantly enhance the mechanical strength and barrier properties of biopolymer-based packaging, offsetting the inherent deficiencies of biopolymers, but also serve as “functional carriers” to endow packaging with active preservation capabilities (e.g., antimicrobial, antioxidant, and ethylene scavenging properties). Additionally, they enable breakthroughs in intelligent functionalities such as freshness monitoring and time–temperature indication. Moreover, nanocomposite packaging constructed using nanomaterials and biodegradable substrates provides a viable solution for achieving high-performance, fully bio-based, and eco-friendly food packaging. This approach proactively addresses the global common concerns of plastic pollution control and sustainable development.

Although nanotechnology exhibits enormous potential in active food packaging, its practical implementation still faces numerous unresolved challenges. Future research should prioritize the safety and environmental compatibility of nanomaterials. On one hand, it is essential to deepen systematic toxicological investigations and develop more sensitive detection methodologies to accurately analyze the migration behavior of nanomaterials in complex food simulant systems, thereby clarifying their potential long-term health impacts on humans. On the other hand, the adoption of “non-migratory” material designs and the widespread utilization of Generally Recognized as Safe (GRAS)-certified nanomaterials will serve as key breakthroughs in ensuring application safety. Meanwhile, the research, development, and application of nanomaterials must strictly comply with green chemistry principles and circular economy requirements. While ensuring that active packaging is fully biodegradable, the synthesis process of nanomaterials should achieve low energy consumption and zero pollution. This strategy prevents technological advancements from imposing additional burdens on the ecological environment, thereby truly realizing the dual goals of “high performance” and “sustainability.”

## Figures and Tables

**Table 1:** Research on different organic nanomaterials in food packaging.

Material Type	Typical Examples	Functional Mechanisms	Application Scenarios
Whey protein	Nanofibrils [47]	Hydrogen bond self-assembly for loading antioxidants	Fruit and vegetable preservation film
Zein	Nanoparticles [48]	Encapsulating non-polar bioactive compounds	Fruit and meat preservation coating
Chitosan	Nanoparticles [49]	Encapsulation of active substances through self-assembly	Meat preservation film
Nanocellulose	Nanoemulsions [50]	As relevant carriers of aroma compounds	High barrier packaging
Starch	Nanoparticles [51]	The high surface area provides adsorption sites, and the hemiacetal structure can undergo nucleophilic interaction with hydroxyl groups	Seafood preservation packaging, short-term food packaging
Nano-liposomes	Nanocomposite coating [52]	Phospholipid bilayer encapsulating antioxidants	Vegetable preservation packaging
Solid Lipid Nanoparticles	Nanoparticles [53]	High surface area behavior, higher diffusion rate in the film matrix and better transmission performance due to its low viscosity	Fruit and vegetable preservation film
Tea polyphenol	Nanocomposite coating [54]	The nanocomposite formed through the interactions between polyphenols and metals can effectively coat the surfaces of various biological substrates	Fruit and meat preservation coating
Carvacrol	Nanoparticles [55]	Phenolic hydroxyl groups interfere with microbial metabolism and have antioxidant properties	Fruit preservation film

**Table 2:** Comparison of differences among inorganic nanomaterials.

	Metal-Based	Metal-Compound-Based	Non-Metallic-Based
Typical example	AgNPs, CuNPs, AuNPs	ZnONPs, TiO_2_NPs, CuONPs, MgONPs	Carbon quantum dots (CDs), SiNPs, SeNPs
Core function	Highly effective antibacterial	Antibacterial + UV shielding/Mechanical reinforcement	Physical barrier + Mechanical reinforcement
Safety comparison	Strictly control the concentration to avoid toxicity	Safe at low dosage levels	The migration risk is the lowest.
Cost comparison	High	Moderate and controllable	Low
Applicable scene	Short-term preservation of high-end foods such as seafood	Low-cost antibacterial packaging such as inner films for ready-to-eat foods, and preservation of alkaline foods such as bread and pastries	High-barrier packaging for oil-based foods such as potato chips and edible oil, and packaging for high-humidity environments to preserve fresh produce

**Table 3:** Effects of different metal-based nanomaterials on biopolymer packaging.

Biopolymer Packaging Materials	Metal-Based Nanomaterials	Impact on Food Packaging
Alginate-Gelatin [58]	AgNPs derived from citrus peel	The film exhibits reduced water solubility and enhanced antimicrobial properties.
Cassava starch [59]	Synthesis of AgNPs Using Basil Extract	The mechanical properties of the film have been enhanced, with improved oxidation resistance, UV protection, and antimicrobial performance.
Pectin-Gelatin [60]	AgNPs loaded with tannic acid	The water vapor permeability of the film has been reduced, its hydrophobicity enhanced, and its oxidation resistance and antibacterial properties improved.
Pectin-Gelatin [61]	AgNPs Loaded with Curcumin	The film exhibits enhanced antioxidant properties, mechanical strength, hydrophobicity, and antimicrobial capability.
Gelatin-corn gluten [62]	Green Synthesis of AgNPs Using Lysozyme	The average diameter of nanofibers decreases, mechanical properties are enhanced, and hydrophobicity and oxidation resistance are improved.
Quinoa Starch [40]	AuNPs	Reduced film permeability, enhanced thermal stability, and improved antibacterial activity against *Escherichia coli*.
Polyvinyl alcohol [63]	AuNPs	The water vapor transmission rate of the film is reduced, the structural stability of the composite material is enhanced, the mechanical properties of the film are improved, and its antimicrobial capability is strengthened.
Carrageenan [64]	Argemone maxicana Leaf Extract-Synthesized CuNPs	The tensile strength of the film increases, the thermal degradation temperature rises, the permeability decreases, the degradability remains unchanged, and the antibacterial properties are enhanced.
Cellulose acetate-polycaprolactone [65]	CuNPs synthesized in Terreus fungal filtrates	The film exhibits enhanced hydrophobicity, increased tensile strength, and reduced water vapor permeability; it also demonstrates improved antimicrobial properties and is non-cytotoxic.

## Data Availability

Not applicable.
